# SARS-CoV-2 origin, myths and diagnostic technology developments

**DOI:** 10.1186/s43042-022-00255-3

**Published:** 2022-03-04

**Authors:** Josephine Wambani, Patrick Okoth

**Affiliations:** 1 KEMRI HIV Laboratory, Kenya Medical Research Institute [KEMRI]-Alupe, P. O BOX 3-50400, Busia, Kenya; 2 Department of Biological Sciences, School of Natural Sciences, Masinde Muliro University of Science and Technology, P. O BOX 190, Kakamega, 50100 Kenya

**Keywords:** COVID-19, SARS-CoV-2, ELISA, RT-PCR, LAMP, TMA, CRISPR

## Abstract

**Background:**

After the first case of COVID-19 being announced in China in December 2019, various diagnostic technologies have been developed at unprecedented pace with the aim of providing a basis for accurate clinical intervention. However, some assays including CRISPR-based diagnostics and loop-mediated isothermal amplification (LAMP) have been less explored. As new COVID-19 technologies emerge, there is need for them to be assessed, validated and improved upon. Moreover, there is paucity of data on the essential factors governing the selection of an appropriate diagnostic approach within the correct timeframe. Myths and origin of SARS-CoV-2 remain to be controversial. Consequently, this review aims at exploring the current COVID-19 diagnostic technologies, performance evaluation, principles, suitability, specificity, sensitivity, successes and challenges of the technologies for laboratory and bedside testing.

**Main Body:**

To date, there exist more publications on COVID-19 diagnostics as compared to the Zika virus. The SARS-CoV-2 virus genome profiles were readily available by 31st of December 2019. This was attributed to the fast-paced sharing of the epidemiological and diagnostics data of COVID-19. Timely profiling of the virus genome accelerated the development of diagnostic technologies. Furthermore, the rapid publication of studies that evaluated several diagnostic methods available provided baseline information on how the various technologies work and paved way for development of novel technologies.

**Conclusion:**

Up to date, RT-PCR is the most preferred as compared to the other assays. This is despite the repeated false negatives reported in many of the study findings. Considering that COVID-19 has caused devastating effects on the economy, healthcare systems, agriculture and culture, timely and accurate detection of the virus is paramount in the provision of targeted therapy hence reducing chances of drug resistance, increased treatment costs and morbidity. However, information on the origin of SARS-CoV-2 still remains elusive. Furthermore, knowledge and perception of the patients toward management of SARS-CoV-2 are also paramount to proper diagnosis and management of the pandemic. Future implications of the misperceptions are that they may lead to increased non-compliance to SARS-CoV-2-related World Health Organization (WHO) policies and guidelines.

## Background

It is evident that COVID-19 pandemic exposed the weaknesses of the healthcare systems worldwide in approaching and addressing the challenges brought about by a global pandemic. The pandemic caused social, economic, financial and healthcare distress globally [1, 2]. Data show that effective and appropriate viral diagnostic testing is necessary for the management and control of the pandemic globally. There is need for assessment, evaluation and improvement of the methods currently available for diagnosing SARS-CoV-2. Accurate assessment of the methods and instruments currently available will provide baseline data useful in the diagnosis of future pandemics [3]. Furthermore, factors governing the selection of an appropriate diagnostic approach within the correct timing are not well defined as limited data in relation to the concept exists. This article reviewed the origin, myths of SARS-CoV-2, mechanisms of virus transmission, principle, suitability, accuracy, successes and challenges of the available SARS-CoV-2 diagnostic technologies.

To date, reverse transcription-polymerase chain reaction continues to stand out as the most accepted technology for SARS-CoV-2 [4]. For this assay to give accurate, reliable and reproducible results, preanalytical, analytical and post-analytical factors are essential, that is complete adherence to the correct procedure for samples collection, handling and transportation, reagents preparation and storage and results interpretation. Improved primer and probe design also enhances the specificity and accuracy of the assays [5, 6]. Other molecular diagnostics are being used but to a lesser degree. This includes the loop-mediated isothermal amplification (LAMP), amplicon-based metagenomic sequencing, immunological assays, CRISPR-based assays, transcriptase-mediated amplification (TMA) and micro-array hybridization-based assays [5, 7–10].

The assays have distinct advantages ranging from the cost of the tests, specificity and accuracy of the assays and reduced turnaround time. The available antigen and antibody testing technology is lucrative in the SARS-CoV-2 population wide screening despite the fact they were initially reported to have low specificity and sensitivity [6, 11, 12]. The technology has the ability of diagnosing a current infection and detecting previous exposure to the virus within a short time hence decisions can be made promptly. Irrespective of the assay employed, repetitive testing is recommended for patients who present with viral pneumonia symptoms or previous history of exposure to the virus [7].

## COVID-19 crisis

COVID-19 has claimed a lot of lives globally [13]. It is the third coronavirus-related outbreak after the SARS-CoV that was announced in 2002 and MERS-CoV in 2012. First case was announced in Wuhan, China, in December 2019 with symptoms of fever, shortness of breath, cough and cold [13–15]. Declaration of more than 97 million infections happened on 20th of January 2021 with at least 1.4 million mortalities leading to SARS-COV-2 being declared a global pandemic [15, 16]. Ten Viral genome sequences were available immediately after the identification of the causative agent of COVID-19 infection and the sequence data revealed 99.98% identity between the genomes [16].

Searches in the database revealed that bat-SL-CoVZC45 and SL-CoVZXC21 strains had the highest proximity to the sequenced data from SARS-CoV-2. The genomes had 88% similarity with SARS-CoV-2 [15]. SARS-CoV-2 genome profiles exposed the similarity that existed between the Virus and SARS-CoV (79%) and MERS-CoV (50%) [15]. With the above similarities, researchers reached a consensus that SARS-CoV-2 might have originated from bats to human beings [15]. Due to the increased spread and fatality rate associated with the COVID-19 disease the scientific community had to come up with highly reliable methods for the diagnosis of the disease with the ultimate goal of providing a basis for accurate clinical intervention [6].

## Mechanism of action of SARS-Cov-2

The virus invades cells by attaching itself to the angiotensin-converting enzyme 2 (ACE 2) on the cell membranes resulting in inflammation and immune reactions [17, 18]. This is usually indicated by increased levels of cytokines, decreased lymphocytes count and damage of body organs [19]. The clinical manifestation of the virus keeps on fluctuating from one person to another with symptoms ranging from asymptomatic cases to symptomatic cases manifesting with fever, shortness of breath, loss of smell and multi-organ failure hence challenging the accurate diagnosis of the virus among different individuals. Therefore, to completely curb the pandemic, there is need for a synergistic approach involving timely accurate diagnosis, monitoring, treatment and prevention of the virus [20]. Since the virus has the capacity to have an effect on the entire body systems, exemplary patients’ management involving diagnosis, monitoring, treatment and prevention of other infections is essential [19]. The first RT-PCR protocol for diagnosing the virus was made available on 23rd January 2020 [13]

## Origin of SARS-CoV-2

It is necessary for everyone to understand the virus origin and for this to happen timely availability of mapping data on several databases on the virus origin is essential. Availability of these data provides background information to researchers about the pandemic and the information may be used by policy makers to formulate appropriate infection control measures [21]. The actual flow of events resulting in the pandemic should be carefully looked into as the mapping data on the virus origin may inform the policy makers about science policy and if it has to be reviewed. Up to date, there are various natural and synthetic speculative hypotheses for the virus's origin [21]. The currently available data are presumptive and hence no evidence of whether the virus results from the laboratory or from a zoonotic encounter [15].

## Mechanisms of SARS-Cov-2 transmission

**Fig. 1:**
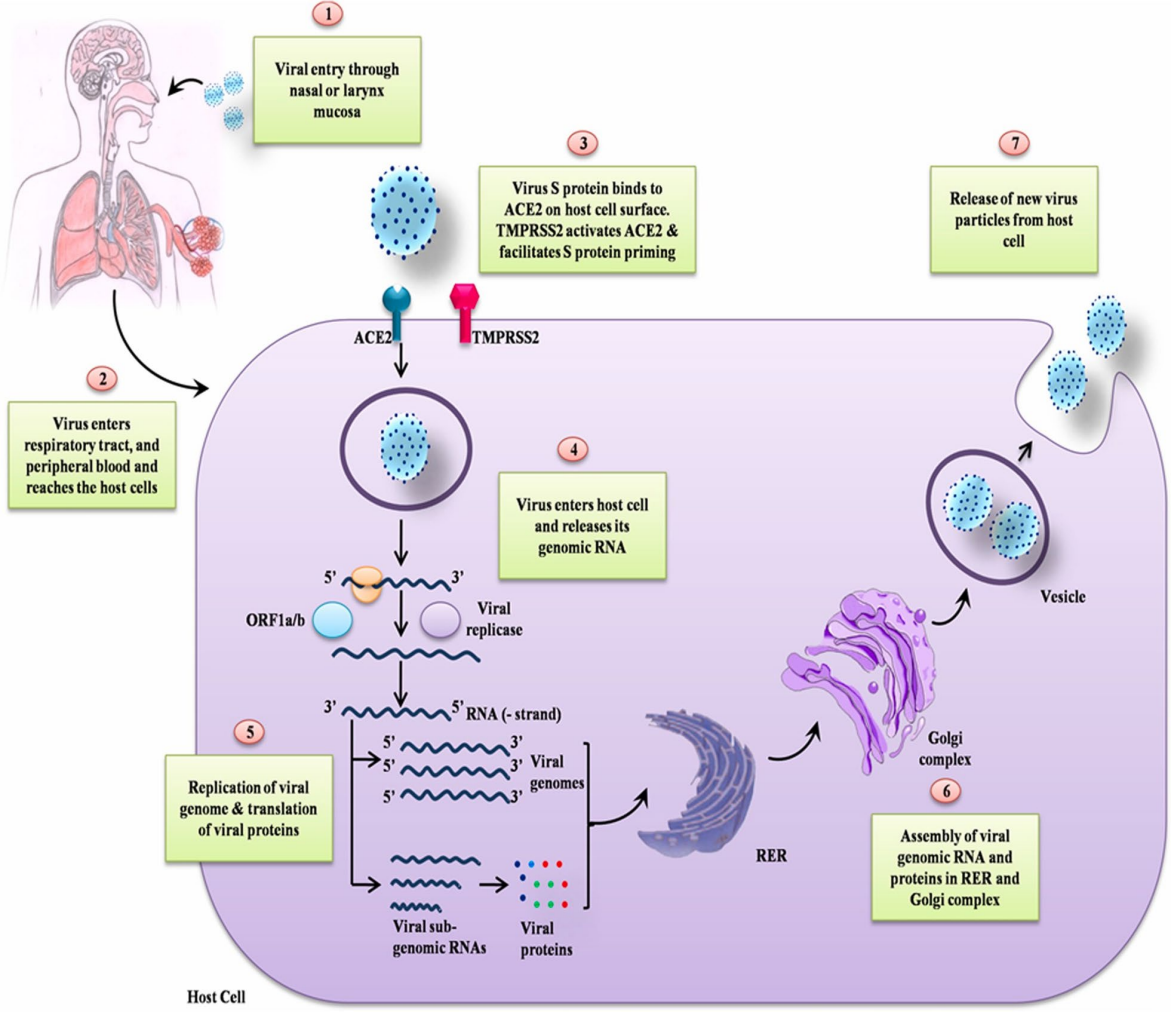
Diagrammatic outline of the SARS-CoV-2 life cycle

SARS-CoV-2 virus gets into the body when one touches the mouth, nose and eyes with virus-contaminated hands. The virus can be accidentally acquired from the environment but for this to happen the temperature and humidity conditions are crucial [12]. Earlier studies suggested that the disease spreads from one human to another through coming into contact with respiratory droplets released from an infected individual while sneezing, talking or coughing [11]. The tiny droplets released while someone is coughing, talking or sneezing have the potential to stay longer in the air and are potential candidates in the spread of the virus from one individual to another. This transmission occurs when the tiny droplets having heavy loads of viruses are inhaled by the healthy individual. The virus is quickly and rapidly spread majorly among close contacts, family members and healthcare personnels [11]. Medical procedures that facilitate the generation of aerosols such as cardiopulmonary resuscitation and intubation have also paved way for the spread of the virus [12]. To combat the spread of the disease, maintaining a safer distance, putting on personal protective equipment and washing hands regularly is highly recommended [22]. What happens is that the SARS-CoV-2 spike glycoprotein attaches to the angiotensin-converting enzyme 2 receptor and enters the host cell [11, 23]. These receptors are found in the kidney, small intestines, lung and heart cells [11]. The virus enters host cells and releases its genomic RNA followed by viral genome replication and subsequent translation of viral proteins. Viral genomic RNA and proteins are assembled and then the new viral particles are released from host cells to infect next cells[11]. The diagrammatic outline of SARS-COV-2 life cycle is represented in Fig. 1. The life cycle is similar to that of MERS-CoV and SARS-CoV [12].

The virus enters host cells and releases its genomic RNA followed by the viral genome replication and subsequent translation of viral proteins. Viral genomic RNA and proteins are assembled and then the new viral particles are released from host cells to infect next cells [12].

## SARS-COV-2 structure

**Fig. 2:**
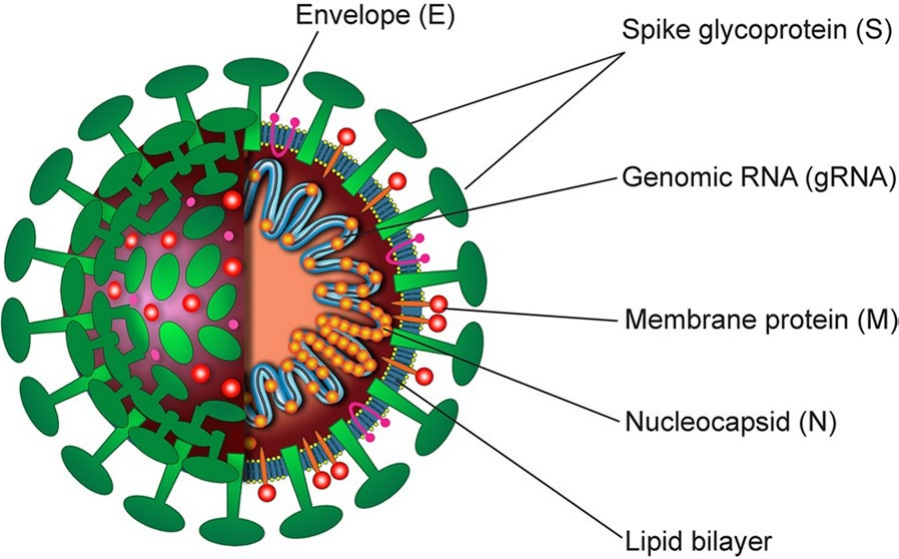
Schematic representation of SARS-CoV-2 structure including the structural proteins. The structural proteins include envelope (E), envelope glycoproteins spike (S), nucleocapsid (N) and transmembrane (M) [22]

Coronaviruses are enveloped, non-segmented positive single-stranded RNA genome viruses belonging to the Coronaviridae family, Nidovirales order [5, 17]. The genome of coronavirus has 6 open reading frame (ORFs) encoding for both structural and accessory proteins within the virus [5]. The N-protein attaches to the genomic RNA forming a capsid. The N-protein is essential in viral assembly, RNA synthesis and translation [5]. Presence of surrounding glycoproteins gives it a crown-like presentation when observed under and electron microscope [11]. Up to date, there exist 5 genera of the virus that has been recognized, i.e., alpha, beta, gamma, delta and Omicron [24]. Delta and gamma infect primarily birds whereas beta and alpha infect mammalian species, Delta and gamma infect primarily birds whereas beta and alpha infect mammalian species [20, 22]. Genes encoding for structural proteins, transmembrane (M), nucleocapsid (N), envelope (E), envelope glycoproteins spike (S) and helicase (Hel) are molecular targets utilized during the PCR process. Additionally, they also have species-specific accessory genes including open reading frame 1a and ORF1b, RNA-dependent RNA polymerase and hemagglutinin-esterase which are crucial in genome maintenance, viral replication, pathogenesis which are crucial in genome maintenance, viral replication, pathogenesis [5, 25]. This is as illustrated in Fig. 2.

Genomic data indicate that ~ 79.5% similarity exists between SARS-CoV-2 and both MERS-CoV and SARS-COV. For the bat coronaviruses, the sequence similarity stood at ~ 96%. As a result, it is likely that the virus was spread and transmitted from bats to humans. The suspected intermediary animal host is either a dog or a pangolin [12].

## Myths about SARS-CoV-2

Science begins with myths and researchers have to come up with complete data to dispute the myths. For SARS-CoV-2 several myths in relation to the disease exist worldwide. Behind each and every science there exists some myths, rationalism, needs, experiments, verifications, validations, approvals and results that should be reviewed. Scientists and researchers are working tirelessly to provide logical arguments in favor or contradiction of the available myths globally. As of July 2020, over 27 myths were already in existence, revolving around SARS-CoV-2 origin, transmission, monitoring, prevention, treatment and management. This included:

## Myths based on origin of SARS-Cov-2

Despite the widely published reports on origin of SARS-CoV-2, a number of misconceptions have been reported as follows:

## SARS-CoV-2 as a zoonotic infection

Medical reports strongly implied that the virus was most likely a zoonotic infection despite the few speculative data already available in the databases [26]. However, reports available revealed that SARS-CoV-2 could have possibly come up as a result of several laboratory recombination processes. These claims were strongly opposed by scientific data which indicated that the 1378 bp (fragment) in SARS-CoV-2 was predominantly distributed in naturally existing coronaviruses, and as a result the source of the virus was less likely to be the laboratory [27, 28].

A publication in the year 2020 clearly highlighted the need for identifying the animal reservoirs for SARS-CoV-2 responsible for the virus spreading from one host to another [29]. Existing data indicated that pangolin species were the reservoir hosts for SARS-CoV-2 and the virus could have possibly spread from the pangolins to humans through humans’ exposure to the wet market available at Wuhan that was involved in the marketing of mammalian species to humans. As a result of enormous diversity of viruses in wildlife, the authors recommended that for there to be a reduction in the future outbreaks there is need for human exposure to animal pathogens to be minimized [30].

Furthermore, data on bats strongly supported the notion that they were possible reservoir hosts for the virus though their intermediate host was not yet defined [31]. These data were strongly backed up by data from a different article that indicated that the SARS-CoV-2 genome had closer proximity to SARS-CoVs from bats and its receptor binding domain had distinct similarities with the pangolin viruses [32]. Further analysis of the virus S gene revealed that possibly the virus could have come up as a result of natural evolutionary events involving a bat-CoV and a pangolin-CoV. New genomic data of SARS-CoV-2 virus showed that the virus was not brought about by the laboratory recombinant processes [33].

Research conducted revealed that 4% variability existed in the genome sequences of SARS-CoV-2 and SARSr-CoV; RaTG13, with notable differences at neutral sites of 17%. Results from a study that was conducted implied that natural selection besides recombination processes could have contributed to the evolution of variations in the receptor binding domain and the functional sites [34]. Exploration of the zoonotic sources of the virus using the existing viruses in Rhinolophid bats and pangolins as a reference indicated that bats and pangolins are highly susceptible to SARS-CoV-2 virus as compared to domestic animals [35].

When the pandemic started, a greater proportion of the COVID-19 cases was directly linked to the wet market in Wuhan selling mammals to humans. The greater similarity that exists between SARS-CoV-2 and bat SARS-CoV suggests that Bats could be the possible reservoir host for the virus [21]. Also, existing data show that the Malayan pangolins (Manis javanica) have distinct similarities to SARS-CoV-2. Scientific data available clearly point out that the virus could have come up possibly through natural selection and subsequent zoonotic transfer [21]. The author also claimed that the SARS-CoV-2 genome could have developed better survival mechanisms during human-to-human transmission [21]. Review data indicated that the illegal trade of wild animals including pangolins could have contributed to the origin of SARS-CoV-2 in the Wuhan City [36].

## SARS-CoV-2 as a laboratory engineered virus

Little scientific information exists to support this hypothesis. Optimization of the receptor binding domain of SARS-CoV-2 to bind human cells and subsequent absence of a furin cleavage site in the viruses of the same group is highly attributed to the laboratory manipulation technologies. Moreover, the simultaneous possession of these two unique features by SARS-CoV-2 is likely to be due to a combination of a natural process and animal serial passage processes [37]. From the data, it is evident that SARS-CoV-2 artificial origin cannot be blindly ruled out. The author emphasized that special attention should be given to the coronaviruses generated in the virology laboratory despite lack of published scientific literature on the same [37]. After closer analysis of the SARS-CoV-2 unique features, the authors concluded that despite the fact the virus could have originated from a natural source, the artificial source was to be given more attention and the search for the potential host in nature was to continue [37].

Published data revealed that SARS-CoV-2 could have possibly emerged in the virology laboratory as a result of routine culturing activities or experiments involving gain of function [38]. Possibly it is in the laboratory that this virus developed mechanisms to successfully attach to human cells resulting in cryptic illnesses among the workers who later spread the infection to the entire population [38].

In conclusion, SARS-CoV-2 origin still remains to be unclear as the data available are only speculative. Genomic variations as a result of repetitive mutations of the virus should be taken into consideration as these mutations may either hamper or facilitate the process of identifying the potential origin of SARS-CoV-2.

## Myths based on transmission

Transmission of SARS-CoV-2 has attracted attention from many researchers. However, other modes of transmission highly publicized have also been widely communicated. They are not supported by scientific evidence and little data are available to support their basis. They include:

## SARS-Cov-2 transmission through houseflies

Cockroaches and house flies may have the capabilities of transmitting the virus by coming into contact with cough droplets from infected persons or contaminated objects or surfaces [39]. These insects feed on remains and residues including animal wastes, blood, nasal secretions, corpses, sputum, pus and human wastes [40, 41] These residues may contain potential harmful viruses and bacteria which are carried on insect body hairs, mouthparts and legs resulting in the spread of infection from one individual to another [40, 41].

## Risk of reinfection

Arguments exist in relation to whether the antibodies produced during the first infection attack offer everlasting assurance on the chances of reinfection. According to WHO, this is not the case as the positive antibodies may not be able to protect an individual from a second viral attack and people should always be cautious despite the fact the second wave of the infection may be mild [41].

## Air conditioning

With air conditioner (AC) social distancing is less useful as the equipment's blown air can facilitate the spread of the virus from one individual to another within a single room. The lower AC temperatures favor the growth and spread of the virus as the air circulates within the rooms. Virus droplets on the floor are blown up by the AC system putting people in the room at a higher risk of contracting the virus. Scientific data indicate that generally infection rate and death rate is comparatively higher among the healthcare professionals possibly as a result of this [41].

## Temperature

Different statistics indicate that there is an association that exists between temperature and COVID-19. Worldometer’s data indicate that countries in warmer regions report significantly lower death rates as compared to countries in colder regions. It seems that the most vulnerable temperature is 10–20 °C [39, 43]. Below this temperature, observations indicate that disease spread is directly proportional to temperature. This is based on the logic that at higher temperatures SARS-CoV-2 virus is inactivated while at lower temperature the virus is able to survive for longer durations on contaminated surfaces [41, 43, 44]. Just like the other viruses, SARS-CoV-2 is highly sensitive to temperature [42]. Studies indicate that temperature affects the ability of the virus to persist and remain viable on surfaces and in air [39, 43].

## Frequent showering with hot water destroys the virus

Developing countries have the notion that showering with hot water frequently is able to destroy the virus. But according to WHO, this is not true as frequent baths with hot water are likely to result in skin burns and cold water may create a conducive environment for the growth and subsequent transmission of the virus hence worsening the situation [39, 41].

## Myths based on management of SARS-CoV-2

There have been wide discussions on the development of vaccines and SARS-CoV-2 management with respect to the pandemic. Many other options and solutions have been recommended besides the WHO and Food and Drug Administration (FDA) recommendations. Some of the recommendations include:

## Consumption of warm water containing ginger and garlic mixture

Traditionally, ginger has proven to be an effective remedy for indigestion, bloating, sore throat and nausea. Garlic on the other hand has higher sulfur content, anti-inflammatory and antiviral properties. Consumption of this mixture results in improved immunity. Due to these scientific findings, garlic is considered as an effective remedy in the reduction of nausea, aiding digestion and fighting flu and asthma, cold and menstrual pains [41]. The available scientific data indicate that consumption of warm water containing garlic and ginger mixture is able to boost immunity resulting in better patient outcomes. The drink has been recommended by the WHO for symptomatic patients as the concoction can boost the immunity of the patient making him/her respond better to the current infection. However, up to date, there exists no scientific evidence that this concoction is a life-saving therapy for COVID-19 infection [39, 41, 42].

## Application of bleach and insecticides on food

Reports indicate that bleach and insecticides when used correctly are effective in destroying the SARS-CoV-2 virus though how it is used and where it is applied remains to be a challenge. The WHO recommended the use of soap, hydrogen peroxide, detergents and bleach as one of the measures to curb the spread of the pandemic. Some of these products are primarily used for human body sanitization while others for household cleaning and fumigation [41]. Instead of using bleach and insecticides, the best practice could be maintaining hygiene in kitchens and storage areas for food at all times [42]. Since these insecticides and bleaches come with a lot of challenges that outweigh the benefits, the WHO recommends that people should utilize the normal methods of washing vegetables and raw fruits and if they must use them, care should be taken to ensure they do not swallow or inhale the chemicals [39, 41, 42].

## Drinking alcohol

Despite the fact that alcohol is one of the ingredients in most of the hand sanitizers and has the capacity of killing SARS-CoV-2 virus on surfaces, there are no data supporting the notion that drinking alcohol provides permanent cure to the COVID-19 infection [42]. Continuous alcohol consumption may aggravate the severity of the illness for the COVID-19 patients.

After the COVID-19 epidemic, there was a famous hoax that alcohol consumption could kill the virus which was spread in the news platforms worldwide and some people blindly adopted it [41]. In Iran, a group of opportunistic people added ethanol to methanol and they distributed the intoxicated liquor to the people. This resulted in Iran hospitals encountering about 700 poisoning deaths at the end of April 2020 [39, 41, 42].

## Ability to successfully hold breath for a period of 10 s

This technique has been employed worldwide to confirm the presence or absence of COVID-19 infection. The inability to hold breath for 10 s and more is associated with COVID-19 infection. Scientifically, individuals whose lung and heart are functioning optimally are able to hold their breath for this period without any discomfort [41]. Data captured disputed the notion as patients infected with the virus could comfortably do this [41].

## Use of ultraviolet ray

Human exposure to ultraviolet (UV) ray is so dangerous as the UV light has devastating effects on humans as compared to the advantages. As a result, WHO recommends the use of soap and water to wash hands rather than going the UV route. Data from researchers indicate that UV light is effective in the disinfection of the virus on the surfaces and has sterility of up to 99.9% [41, 43].

## Use of mustard oil

It is believed that application of mustard oil in the nostrils is effective for the complete elimination of SARS-CoV-2 virus from the body via the stomach. The oil contains selenium, fatty acids, magnesium and antiviral elements. The oil is an effective remedy in the fight against cold and bacterial attack. To date, there is no scientific proof that application of mustard oil in either nostrils or body parts offers protection against SARS-CoV-2 [41].

## Pneumonia vaccine

Since SARS-CoV-2 and Pneumonia have some overlapping signs and symptoms people felt that the pneumonia vaccine could work magic. The vaccine has Streptococcus pneumoniae bacterium that protects the lungs during severe pneumonia attacks. Data from different researchers illustrated that pneumonia vaccinated individuals have better protection against SARS-CoV-2 virus [41, 42]. However, the WHO recommends the application of any therapy that can boost the respiratory system making it function optimally. SARS-CoV-2 is a new strain with complex dynamics which are completely different from the other pathogens. To date, there is no evidence that the pneumonia vaccine offers protection against the SARS-CoV-2 virus [42].

## Use of antibiotics

WHO does not recommend use of antibiotics since these antibiotics may kill the normal flora worsening the already bad situation [41]. SARS-CoV-2 is a virus and not a bacterium. Antibiotics work effectively against bacteria and not viruses. Most viruses have no cure and diagnosis, monitoring and prevention are the only key alternatives. Since SARS-CoV-2 is not a bacterium, it is undoubtful that the antibiotics cannot be used as means of prevention and treatment. Antibiotics should only be administered to cure underlying opportunistic infections [42].

## Diagnosis of COVID-19

Laboratory tests including molecular assays, serological assays, radiological assays coupled with clinical findings are important in COVID-19 diagnosis [11]. The available approaches work by detecting the presence of the virus or the antibodies produced against the viral infection [7, 11, 45–47]. Samples are collected from the lower and upper respiratory regions through swabs for molecular testing [12]. Blood and urine can also be used though they are not useful clinical specimens since at a times the virus goes undetected [12]. Studies have indicated that the sputum samples and bronchoalveolar lavage are the recommended samples as they have higher viral loads as compared to the other specimens [25].

In research, the quality of results generated depends on the quality of preanalytical, analytical and post-analytical processes [12]. The same applies to COVID-19 testing in which proper respiratory tract specimen collection at the right anatomical site at the right time by qualified personnel is essential for accurate laboratory diagnosis of the infection. The sample should be collected and transported in the appropriate Transport Medium and refrigerated at the right conditions prior to testing. During testing, the integrity of the samples and reagents should be retained and proper attention should be given to how the results are interpreted. Complete compliance to the mentioned stages is crucial for the generation of results that are accurate, reliable and reproducible [25].

Molecular technologies scan for various markers such as RNA-dependent RNA polymerase, specific sites of the viral genome and the structural proteins [12, 48]. At the moment, different technologies approved by regulatory agencies globally for the systematic, timely diagnosis of COVID-19 at different stages are available [6]. Several factors should be put into consideration when selecting a diagnostic method to be utilized and they include sensitivity and specificity of the technology, turnaround time and cost of the detection assay. Finally, before a diagnostic technology is selected, certain factors including the purpose of the investigation and how often the test will be conducted must be put into consideration [6].

**Fig. 3:**
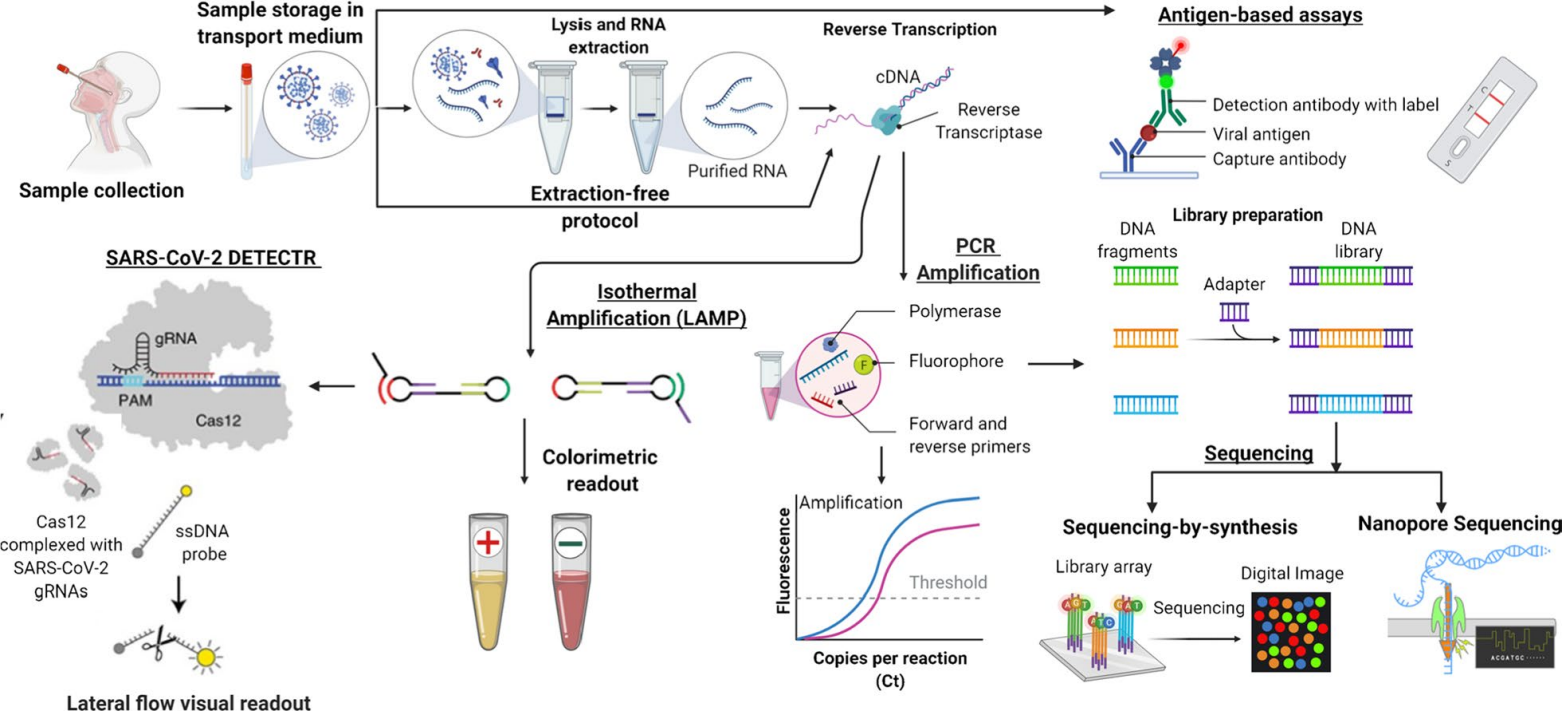
Schematic sketch of COVID-19 diagnostic process. Respiratory specimens collected and kept in appropriate transport. During samples extraction cells are lysed and RNA is extracted, amplified and detected by either PCR or isothermal route. Detection is by either use of specific fluorescent dyes or a calorimeter. For CRISPR-based technology, the activated Cas12 cleaves reporter labels resulting in generation of a fluorescent signal which is detected and quantified. In the sequencing process, the cDNA is converted into a form compatible followed by detection of these cDNA sequences digitally. The antigen tests scans for the presence of SARS-CoV-2 antigens whereas the antibody tests scans for the presence of antibodies produced against the virus [49]

Currently, the available diagnostic assays are classified into 3 broad groups which are composed of the Molecular tests, serological assays and Radiological assays [6]. Among all the diagnostic technologies for SARS-CoV-2, molecular tests and serological tests are preferred [11]. Figure 3 illustrates the COVID-19 workflow followed by step-by-step explanation of the molecular diagnostic approaches and immunological assays.

## Reverse transcription-polymerase chain reaction

This technology is the most accepted technology and is used as a reference in the diagnosis of SARS-COV-2 in all the clinical samples ranging from oropharyngeal swabs, nasopharyngeal swabs, serum, stool, ocular secretions, saliva among others [4, 5]. Diagnostic laboratories are currently having difficulty detecting the new Omicron variant. The diagnostic performance of available PCR assays is currently unknown, as all information available at this early stage is based on companies' in silico evaluations. There isn't much real-world evidence for many of the systems yet. To quickly assess the performance of the available systems, laboratories should share RNA extracts from positive cases [50].

The technology has several major steps which are denaturation, annealing and elongation which happens at 95 °C, 50 °C and 72 °C, respectively [4, 5]. The Viral RNA is first converted to cDNA followed by annealing of primers to the specific sequences. Detection is achieved by the use of specific probes. The RT-PCR technology checks for the presence of E gene followed by targeting the RdRp gene using specific primers and probes [11]. The limit of detection is 3.9 copies and 3.6 copies for E gene and RdRp gene, respectively. Cycle threshold values less than 37 are interpreted as a positive [8, 12]. The RT-PCR process is tracked real time by specific probes. Different RT-PCR reagent test kits exist but the principle is the same and they target various markers such as RNA-dependent RNA polymerase, specific sites of the viral genome and the structural proteins [48]. The technology is semi-automated, hence reducing the possibility of exposure to infection by the laboratory personnel. This technology comes with numerous challenges including requirement of expensive instruments and highly competent personnel to operate the equipment[11]. Furthermore, it takes a lot of time for the assay to generate results [8, 11].

The technology comes with numerous advantages including the process of amplification, detection and quantification being done in an enclosed system hence ruling out the possibility of false-positive that is usually associated with the amplified product cross-contamination [6, 7, 25]. PCR assays target different regions of genes and hence the results are interpreted differently. For assays targeting two different regions results can be positive, negative or inconclusive. For the inconclusive results, the samples must be re-tested [5].

Study findings from previous analysis however report the challenges of limitation of application of RT-PCR, risk bias issues and high heterogeneity. It is recommended that repeatability of testing of patients with SARS-Cov-2 will solve these cases of false negative RT-PCR findings based on data captured up to July 2020 [51].

## Reverse transcription-loop-mediated isothermal amplification (LAMP)

This technique works by amplifying the viral genomic targets at a constant temperature. The amplified product is then continuously extended, circularized and re-extended resulting in the production of DNA with different stem-loop structures [17]. The technology has the reverse transcription step and uses four specific different primers including the forward inner primer-FIP, a backward inner primer-BIP, outer backward primer-B3 and outer forward primer-F3 which is specific for the target sequence of the genome [12]. Photometry principle is employed in the detection process. It is a very simple method that is less expensive and highly sensitive [5, 11, 52]. It only requires heating and visual monitoring [8, 13]. Turbidometer is used to measure the intensity of color change which is equivalent to the viral content present in the sample [12]. Limitations of the technique are that a well-trained and competent personnel is required to perform the assay. Accuracy of the results is based on the type of samples used and the integrity of the reagents used to perform the assay. Amplification inhibitors and high mutation rates of the virus may alter the integrity of the results generated by the assay [7, 12].

## Transcription-mediated amplification (TMA)

The technology utilizes T7 RNA polymerase and retroviral reverse transcriptase to amplify DNA targets or viral RNA. It requires a single temperature unlike the RT-PCR which requires different temperature cycles. This technology can detect different pathogens present in the same samples. A good example of an instrument that employs this technology and RT-PCR is the Panther equipment from Hologic. It has high specificity and sensitivity with high sample throughput [7, 8].

## Recombinase polymerase amplification

**Fig. 4:**
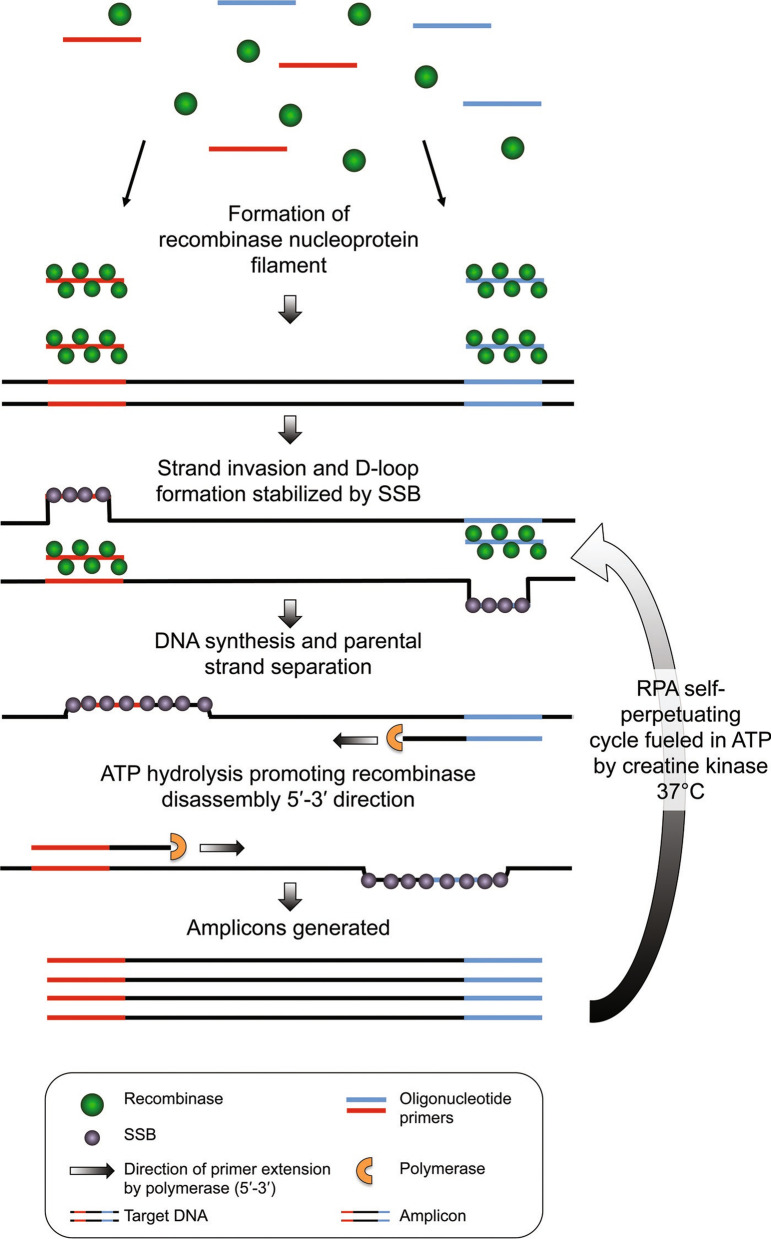
Schematic representation of RPA cycle. The assay is composed of DNA polymerase enzyme, Recombinase enzyme and SSB which facilitate the amplification of specific genes at a single temperature [10]

This technology utilizes recombinase enzyme in the recognition of specific DNA sequences followed by amplification and detection of the viral-specific genes. It operates at a single temperature unlike the RT-PCR. The technique can be used in the clinical diagnosis of COVID-19 infection [8, 53]. It is an affordable, rapid and simple technology that does not require the PCR machine [54]. For a successful RPA, certain intrinsic and extrinsic factors must be adhered to. Background DNA and inhibitors affect the integrity of the results generated. Post-amplification treatment and amplicon clean-up are essential for quality results. Probes, primers and nucleic acid template design affects the specificity and sensitivity of the assay. Carrying out the assay at the right temperature is essential for credible results [9]. The technology utilizes single-strand DNA binding protein and Escherichia coli RecA recombinase to compensate for the denaturation step [10]. During the process, the recombinase enzyme binds to oligonucleotide probes and primers forming a nucleoprotein filament. The formed nucleoprotein filament checks for homologous sequences in the target DNA and once this is identified the nucleoprotein filament invades the target DNA resulting in the formation of D-loop structure. Hybridization of primer onto the target DNA and stabilization of the complementary strand by the Single-Strand DNA binding protein takes place (shown in Fig. 4). ATP hydrolysis induced by RecA protein facilitates the recombinase disassembly process allowing DNA polymerase enzyme to elongate the primers. The generated DNA strands are subjected to repetitive RPA cycles until exponential amplification is achieved [10]. The reaction takes place at a temperature of 37 °C [10]. This is well illustrated in Fig. 4.

## CRISPR-based assays

The CRISPR-Cas13 system is a sequence-specific RNA-sensing tool that has recently been used to create more flexible and simplified testing formats. Because of the limitations of traditional diagnostic procedures such as real-time PCR-based methods and serological tests, scientists developed alternative nucleic acid detection approaches for SARS-CoV-2, addressing the urgent need for more testing. These methods aim to provide fast, accurate, cost-effective, sensitive and high-throughput detection of SARS-CoV-2 RNA on a variety of specimen types without the need for specialized equipment or expertise [55].

Cas9, Cas12 and Cas13 are special CRISPR enzymes which work by recognizing and cutting sequences [13]. Scientific engineering of Cas12 and Cas13 family of CRISPR enzymes allows them to detect and even cleave different viral RNA sequences. Two different tests took advantage of this technology that is the SHERLOCK and DETECTOR test. In the SHERLOCK test, Cas13 cuts the reporter RNA following its activation by the SARS-CoV-2-specific guideline RNA [13]. On the hand, the DETECTR assay utilizes Cas12a to cut the reporter RNA. Recognition of viral RNA sequences of the E and N genes is facilitated by the synthetic SARS-CoV-2 RNA fragments [5, 11]. During the actual process, the viral RNA targets are reverse transcribed using reverse transcriptase enzyme forming cDNA. The cDNA is amplified and transcribed to RNA isothermally. The final RNA fragments combine with Cas13a protein forming a SHERLOCK [12, 13]. The technology has high specificity and sensitivity and does not require the purchase of expensive instruments. Also, the test takes little time for the results to be generated [7, 8].

**Fig. 5:**
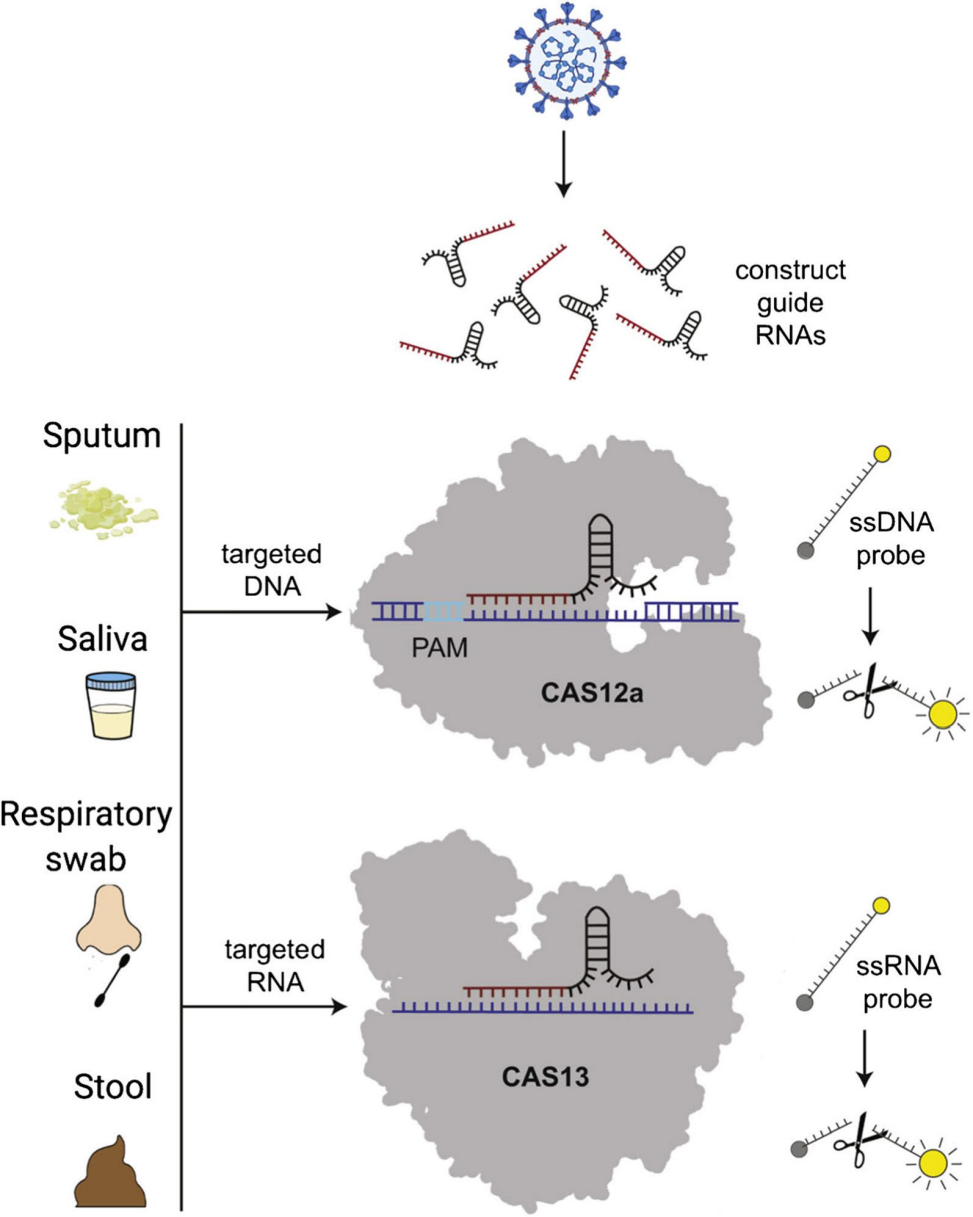
Diagrammatic illustration of CRISPR-based technology [49]

Figure 5 shows the diagrammatic illustration for CRISPR-based assay. The system utilizes a CRISPR-associated nuclease (Cas) and guide RNA (gRNA). The nucleotide sequence in the gRNA is always complementary to the target sequence. Activated Cas13 cuts the reporter RNA sequences resulting in the production of a fluorescent signal which is detected and quantified [49].

## Micro-array: hybridization-based assays

First, viral RNA is converted to cDNA by the help of a reverse transcriptase enzyme. The generated cDNA is then labeled with probes targeting specific cDNAs in the clinical samples. Hybridization of the labeled cDNAs to the complementary oligonucleotide sequences on a chip takes place. After washing the unhybridized cDNAs, the hybridized cDNA signal is then quantified by a detector [11].

The technology has maximum accuracy and is useful in the detection of variant strains of SARS-COV-2 and mutations in the virus genome [8, 11]. Data available indicate that the assay detected mutations in the SARS-CoV gene with 100% accuracy. Rapid identification and detection of mutations are necessary as the generated data serve as a reference for further and future studies [11].

## Amplicon-based metagenomic sequencing

The technology utilizes a dual approach involving use of amplicon-based sequences and metagenomic sequencing [11]. SARS-CoV-2 virus can be identified using metagenomic sequencing. The technique identifies pathogens responsible for the secondary infections worsening the COVID-19 symptoms by providing individuals background microbiome data [11]. It is useful in the identification of SARS-COV-2 mutations and in studies involving recombination events. It has the capability to identify other pathogens present in the sample [8, 11]. This technology was utilized in the sequencing of SARS-CoV-2 genome [11].

## Immunological methods

The focus of epidemic prevention and control has shifted to extensive serological antibody testing of the population to monitor population infection status, vaccine efficacy, immunity persistence, and high-titer neutralizing antibody screening and collection. These tests, such as the enzyme-linked immunosorbent assay (ELISA), chemiluminescent immunoassay (CLIA), immunofluorescent assay (IFA) and colloidal gold immune chromatographic assay (GICA), are based on detecting SARS-CoV-2 via IgM and/or IgG antibodies in serum or body fluid samples [56]. Existence of these techniques has facilitated mass diagnosis of SARS-CoV-2 [47]. ELISA technology utilizes an enzyme conjugated antibody to check for the presence or absence of specific viral antigen or antiviral antibody in clinical samples. Color production indicates a positive result and no color indicates a negative result. The color can be observed visually or by the use of a spectrophotometer. The color intensity is equivalent to the amount of complex formed between the antigen and the antibody [47].

The technology checks for the presence or absence of viral antigens present in the specimen or viral antibodies formed by the body in response to an infection [12]. The method is able to confirm past COVID-19 exposure. The advantage of this technology is that it is not time-consuming to get the results and also it is cost friendly. Also, the test can be performed at the bedside hence decisions are made in time [6].

Finally, for the assay to detect both the active and past infections, the assay must be performed at the appropriate time phase of the infection [5]. However, the results generated are sometimes not very accurate due to poor sensitivity and specificity of the assay in early infections [11, 25]. Furthermore, since the body takes several weeks to produce IgG and IgM responses, this technology may not be essential in the active case management [6, 11, 25].

The antigen-based immunoassays take advantage of SARS-CoV-2 structural proteins, for example the S protein is usually utilized as it is highly immunogenic and also a major transmembrane protein [11]. The assay scans for the presence or absence of Protein E and N and the S protein is utilized as the target as its amino acid sequence is unique facilitating the accurate detection of the virus [11].

**Fig. 6:**
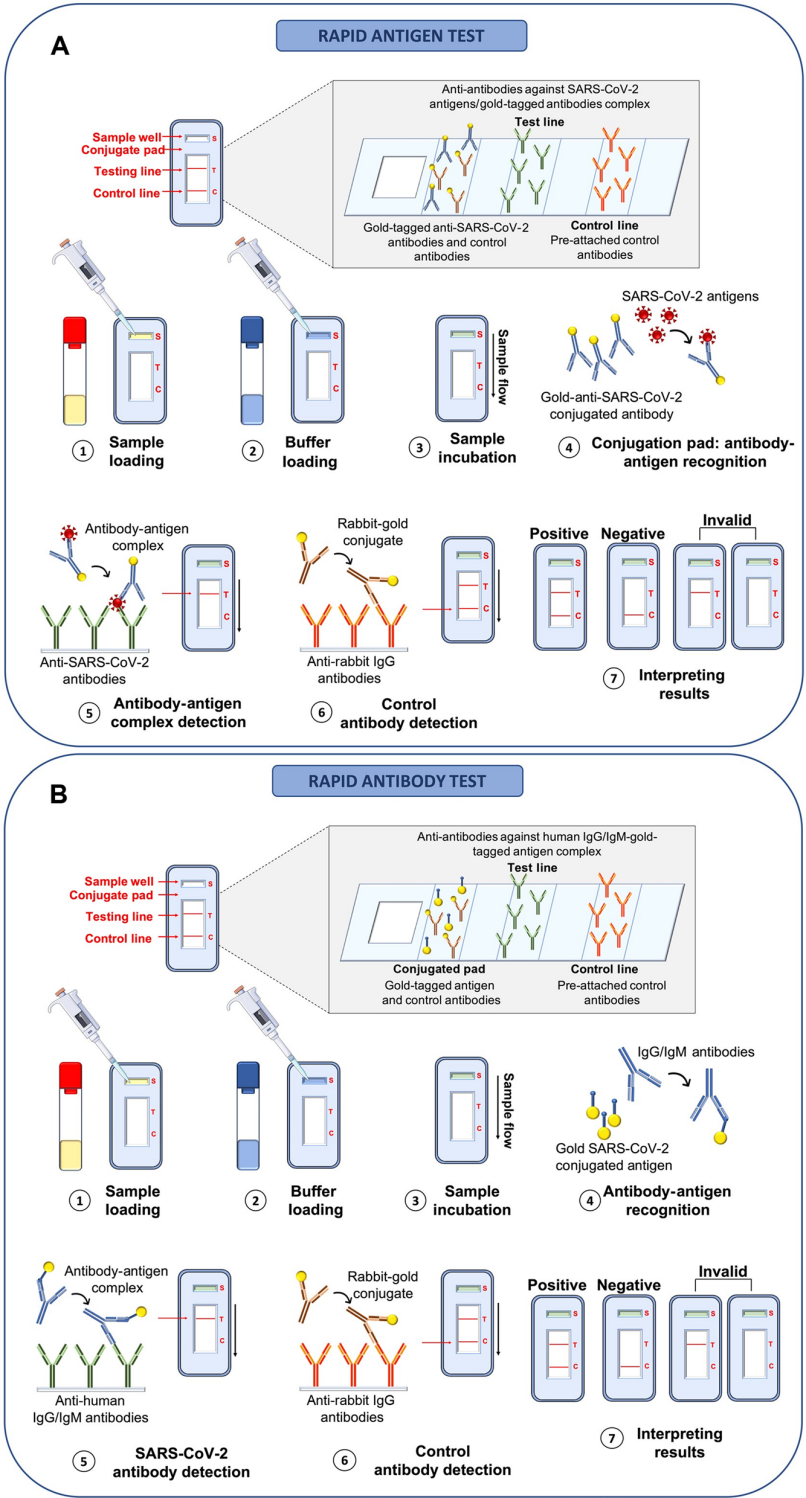
Diagrammatic outline of the antigen testing (**a**) and antibody testing (**b**). A indicates the analytical process for rapid SARS-CoV-2 Antigen detection and B indicates the analytical process for rapid antibody detection of IgA, IgM and IgG against SARS-CoV-2 in clinical specimens [6]

The antibody-based immunoassay checks for the presence or absence of antibodies (IgG and IgM) which are produced by the body at different periods of the infection. The assay results are affected by several parameters including applied test, individual patient variability and viral features. As a result, it is essential for an appropriate assay to be selected within the correct time frame if accurate diagnosis is to be made [7, 11] (Fig. 6).

## Conclusion

In conclusion, despite the scientists determination to uncover the origin of SARS-CoV-2, the information still remains elusive with many perceived conspiracy theories. Furthermore, more myths have erupted, leading to misconceptions and perception of the origin, transmission and management of the SARS-CoV-2. Probably, this may lead to failure to adhere to the WHO and FDA SARS-CoV-2 management strategies. This is likely to derail the gains made on the spread of the disease. In addition, RT-PCR remains to be the gold standard for testing SARS-CoV-2. Immunological methods are useful in the mass testing of the virus since results are obtained in real time. Finally, despite the diagnostic assay employed, for accurate, reliable and reproducible results, the preanalytical, analytical and post-analytical stages are essential. Furthermore, complete adherence to the essential factors governing the selection of an appropriate diagnostic approach within the correct timing is paramount. Regular assessment, validation and improvement of the available diagnostic approaches are necessary for continuous provision of credible results.

## Data Availability

Not applicable.

## Abbreviations

LAMP: Loop-Mediated Isothermal Amplification
TMA: Transcriptase-Mediated Amplification
ACE2: Angiotensin-Converting Enzyme 2
RT-PCR: Reverse Transcription-Polymerase Chain Reaction
UV: Ultraviolet
AC: Air Conditioner
RPA: Recombinase Polymerase Amplification
ELISA: Enzyme-Linked Immunosorbent Assay
WHO: World Health Organization
FDA: Food and Drug Administration
